# Schwannoma of Kidney: A Case Report of a Benign Entity With Rare Presentation

**DOI:** 10.7759/cureus.80789

**Published:** 2025-03-18

**Authors:** Dhivya N, Shilpa Ramasamy, Suganthi K

**Affiliations:** 1 Department of Histopathology and Cytology, Apollo Hospitals, Chennai, IND

**Keywords:** diagnostic dilemma, nerve sheath tumour, renal, schwannoma, spindle cell neoplasm

## Abstract

Schwannomas are rare peripheral nerve sheath tumours involving the head, neck and extremities. Heart, lung, orbit and kidney are some of the rare sites for schwannoma involvement. Here, we present a 55-year-old patient with no specific complaints, who was detected to have a complex solid cystic mass in the lower pole of the left kidney. In contrast-enhanced computed tomography, the lesion was highly suggestive of malignancy. The patient underwent a laparoscopic radical nephrectomy. Grossly, the tumour was well-circumscribed and delineated from surrounding renal parenchyma. Microscopic examination revealed variably cellular spindle cell neoplasm arranged in palisades with intervening myxoid and cystic change. On immunohistochemistry, S100 and SOX10 were diffusely positive, consistent with schwannoma. We report this case for its rarity and to emphasize the fact that clinical and imaging findings are non-specific for this entity as it mimics the common renal neoplasms. Histopathology with supportive immunohistochemistry clinches the diagnosis.

## Introduction

Schwannomas are benign mesenchymal neoplasms arising from the Schwann cells, predominantly involving the head, neck and extremities. Fifty seven percent of schwannomas arise from the vestibulocochlear nerve. Spinal intramedullary extra-dural schwannomas are also common, with multiple paraspinal schwannomas associated with Neurofibromatosis type 2, Carney’s complex or Schwannomatosis [1]. Heart, lung, orbit, salivary gland and vulva were some rare sites in which schwannomas have been reported. The retroperitoneum, particularly the kidney, is one of the rare sites of schwannoma occurrence. Here, we report a case of schwannoma presenting as an incidentally detected renal mass in imaging for its rare site of occurrence, posing a diagnostic challenge from clinical and radiological perspectives.

## Case presentation

**Figure 1:**
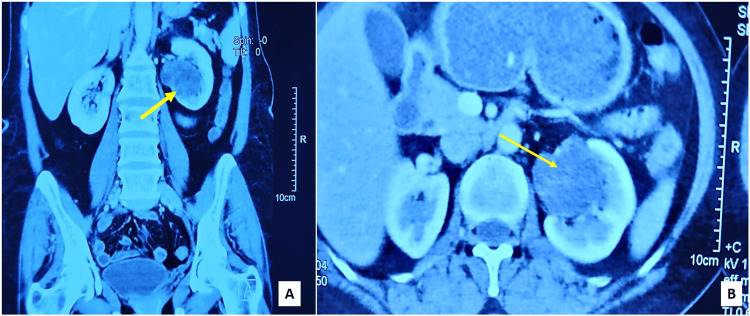
Contrast-enhanced CT KUB. A: Coronal view. B: Axial view. Sections reveal complex solid cystic mass (arrows) in the mid and lower pole of left kidney, distorting the pelvicalyceal system. KUB - Kidney, ureter and bladder

A 55-year-old female underwent a preventive health check, during which a solid cystic lesion measuring 4.3 × 4.1 cm was found in the left kidney by ultrasonogram of the whole abdomen. The patient previously underwent surgery for pituitary macroadenoma and cranioplasty with mesh repair for frontal osteomyelitis at the age of 40 years. At present, she has no specific complaints and no family history. On physical examination, the abdomen was soft and non-tender. No lesions were identifiable elsewhere in the body. The urine cytology was negative for high-grade urothelial carcinoma. Computed tomography with contrast revealed a complex solid cystic mass in the mid and lower pole of the left kidney, measuring 5×4.5×4 cm, distorting the left renal pelvicalyceal system. It was categorized as a Bosniak IV cyst (Figures 1A-1B).

**Figure 2:**
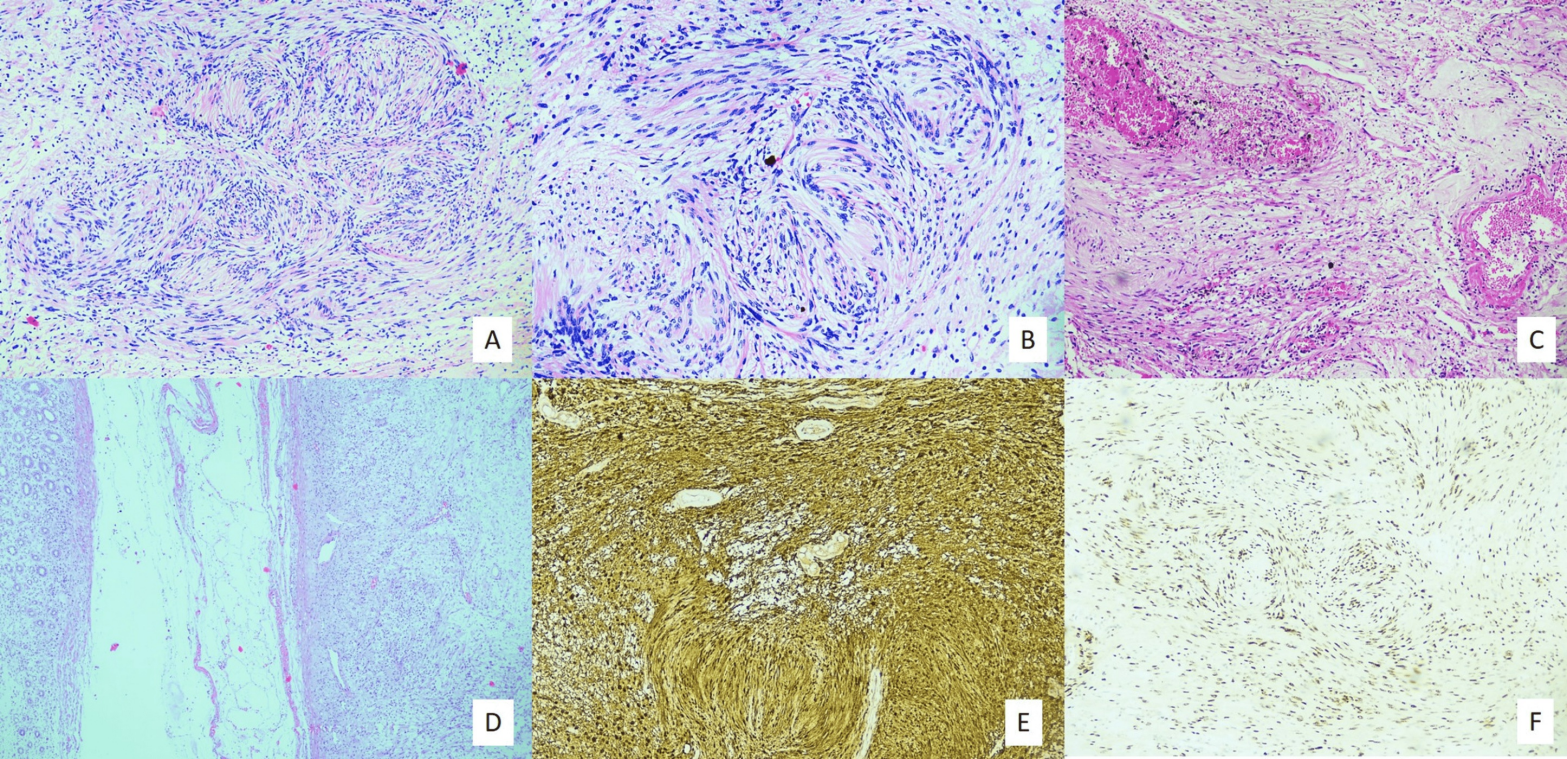
Histopathology and Immunohistochemistry images A (40×) and B (100×): Cellular spindle cell neoplasm arranged in whorls. C (100×): Hyalinized thick-walled blood vessels and hemosiderin deposition in hypocellular areas. D (100×): Encapsulated tumour in close association with renal parenchyma. E (100×): Diffuse S100 staining. F (100×): Strong and diffuse nuclear expression of SOX10.

So, the patient underwent laparoscopic left radical nephrectomy and the specimen was received for histopathological examination. On gross examination, a well-circumscribed greyish tan and pale yellow solid cystic lesion was noted in the lower pole, near the hilar aspect, measuring 4.5×4×3.3 cm. The lesion had sharp demarcation from adjacent renal parenchyma with no gross infiltration into any structures such as renal capsule, perinephric fat, renal pelvis, renal sinus soft tissue, hilar vessels and ureter. On microscopy, there was a well-circumscribed and delineated spindle cell neoplasm (Figures 2D), composed of palisades of neoplastic cells with elongated wavy nuclei and eosinophilic fibrillary cytoplasmic processes arranged in prominent hypercellular and hypocellular patterns (Figures 2A-2B). The hypocellular areas showed myxoid change with interspersed hyalinized blood vessels, areas of cystic changes and pigment-laden macrophages (Figure 2C). There was no marked nuclear pleomorphism, necrosis or mitotic activity. A thorough sampling of the tumour was done to detect epithelial or other elements. The morphological diagnosis considered was spindle cell neoplasm of neural origin. Immunohistochemistry was performed. There was diffuse and strong nuclear and cytoplasmic positivity for S100 (Figure 2E). SOX10 was extensively positive (Figure 2F).

PAX8 and GATA3 were negative, which ruled out primary renal and urothelial neoplasms. Hence, the integrated histopathological and immunohistochemical diagnosis was consistent with the schwannoma of the renal parenchyma. The patient is undergoing regular follow-up and shows no signs or symptoms of the disease.

## Discussion

Schwannomas are benign peripheral nerve sheath tumours. In 1932, Masson coined the term schwannoma [2]. The terms schwannoma and Neurilemmoma have been interchangeably used since then.

Our comprehensive web-based search in the PubMed database using the terms ‘renal’ and ‘Schwannoma’ showed previously reported 48 cases of renal and peri-renal schwannomas in English literature. Renal schwannomas showed female predominance with non-specific symptoms and were detected incidentally in a few cases [3]. The first case of renal schwannoma was reported by Philips et al. in 1955, following which 47 cases were reported to date [4]. All the cases were of adults with an age range of 18-89 years. These indolent neoplasms were associated with compressive symptoms as they grew large. The most common presenting symptoms were abdomen and flank pain, followed by fever with chills and gross haematuria [4]. Similarly, our patient was a female with no specific symptoms and incidental diagnosis. The mean size of the tumour was 7.3 cm. A total of 56.25% of lesions involved right kidney and 43.75% involved left kidney [4].

The most common imaging modalities employed for diagnosis are CT with contrast enhancement and MRI imaging. In MRI, schwannomas are isointense on T1-weighted images and hyperintense on T2-weighted images. Likewise, with gadolinium contrast, there is a homogenous enhancement of the solid component of renal schwannomas on T1-weighted images [5]. However, these modalities are not entirely specific and can lead to diagnostic confusion. Renal schwannomas can mimic other primary renal neoplasms such as renal cell carcinoma, renal pelvis tumours, and solitary fibrous tumours, among others, especially when they exhibit features like haemorrhage and cystic change. Therefore, a comprehensive understanding of the differential diagnoses is crucial in the accurate diagnosis of renal schwannoma.

Performing a needle biopsy had limited utility because of potential pitfalls associated with imaging, such as non-diagnostic samples due to pseudo-enhancement, haemorrhage and protein content mimicking a solid lesion, and fat content of the lesion. Also, 95% of needle biopsies performed for Bosniak cyst IV are malignant - hence, biopsy was not recommended. However, recent advances in imaging have led to highly accurate diagnostic yield, which aids in detecting benign lesions with minimal morbidity, differentiates primary from secondary renal neoplasms, and helps in informed therapeutic decision-making [6].

Most of the cases (85.5%) in the literature underwent either partial or radical nephrectomy. Tumour excision was performed in 14.5% of cases - both partial nephrectomy and tumour excision required complete tumour removal with negative resection margins [7,8]. When the tumours were large, adhered to adjacent structures and radiologically mimicked renal cell carcinoma, a radical nephrectomy was performed. Usually, kidney-preserving surgeries were performed among patients with solitary kidney, bilateral renal neoplasms and renal insufficiency. However, kidney-preserving surgeries are recommended even with normally functioning contralateral kidney [9]. In the present case, radiology showed a complex solid cystic lesion in the lower pole of the left kidney, measuring 5×4.5 cm, mimicking a renal cell carcinoma. Hence, the patient underwent radical nephrectomy.

Renal schwannomas are solitary lesions most located in the renal hilum (41.7%), followed by renal parenchyma (37.5%), renal pelvis and capsule. This distribution was explained by the entry of parasympathetic nerve cells through the renal artery at the renal hilum. On microscopy, the tumours were encapsulated and fairly circumscribed with a light-tan glistening appearance and areas of haemorrhage [10]. Microscopy typically showed Antoni A and Antoni B areas. Antoni A area is defined as compact and highly cellular areas with palisades of tumour cells exhibiting modest eosinophilic cytoplasm, no discernible cell borders, and normochromatic elongated tapered nuclei.

In contrast, Antoni B areas contain a cobweb-like network of tumour processes with collections of lipid-laden histiocytes and thick-walled, hyalinized blood vessels. Subcapsular lymphoid aggregates can be seen. Morphological differential diagnoses included malignant fibrous histiocytoma, solitary fibrous tumour, sarcomatoid renal cell carcinoma, and leiomyoma. On immunohistochemistry, diffuse and strong nuclear and cytoplasmic positivity of S100, along with extensive SOX10 positivity, confirmed the neural crest origin of the tumour.

A causal link exists between schwannoma tumorigenesis and merlin tumour suppressor protein, produced by the *NF2* gene located on chromosome 22q12.2 [11]. NF2-inactivating mutations were noted in sporadic tumours with frameshift and nonsense mutations [12]. None of the cases reported in the literature had Von Recklinghausen disease or extra-renal schwannomas.

The literature reported 44 benign cases, and only 4 malignant cases [4]. The most common metastatic site was the lung, followed by bone, diaphragm, mesentery, and colon [13]. The prognosis of the benign tumours was better, with patients showing no symptoms or signs of the disease at follow-up.

## Conclusions

We present this rare case of renal schwannoma for its presentation at the rare location and posing a diagnostic confusion with malignant renal tumours such as sarcomatoid renal cell carcinoma, and solitary fibrous tumour - however, exhaustive histopathological examination and application of immunohistochemical markers aided in the diagnosis. Hence, with the evidence that malignancies had arisen from these neoplasms, we suggest thorough sampling of the lesion to avoid misdiagnosis. Tumour excision or radical nephrectomy has been the standard treatment modality. Because of their good prognosis at follow-up, kidney-preserving surgeries may be done in future.
